# Insights into coaching a women's national futsal team

**DOI:** 10.3389/fspor.2025.1533224

**Published:** 2025-03-18

**Authors:** Fraser Carson, Tihana Nemčić Bojić, Khatija Bahdur

**Affiliations:** ^1^ Department of Sport, LUNEX, Differdange, Luxembourg; ^2^ Luxembourg Health and Sport Sciences Research Institute A.S.B.L., Differdange, Luxembourg; ^3^ Faculty of Kinesiology, University of Zagreb, Zagreb, Croatia

**Keywords:** high-performance sport, international tournament coaching, team cohesion, team development, coaching behaviors, coach communication

## Abstract

**Introduction:**

Tournament coaching is known to provide different challenges to coaching a team across a regular season. There is limited time to prepare, meaning that team roles and objectives need to be established quickly, and communication must be effective.

**Method:**

This interpretive ethnographic case study explored how the coach of a women's national futsal team approached this, while competing at a week-long tournament in preparation for a World Cup qualification event.

**Results:**

Following the completion of five interviews (one prior to, three during, and one post tournament), thematic coding produced three main themes: (1) Keeping consistency in development and focus, (2) Communicating openly, and (3) Individualizing approaches.

**Discussion:**

The coach established a clear goal for the tournament, with the focus on the team's game plan and ability to implement the tactics they want at the World Cup, which helped create performance targets that were independent of the results to help maintain consistency. A transformative leadership style, underpinned by free communication empowered the players to buy into the system and enhanced motivation and commitment. The coach, with her staff, made a deliberate effort to spend time individually with players, as this allowed players to create autonomy, which enhanced commitment. The findings provide insights for coaching women's teams in general and add further information about coaching in an idiosyncratic tournament environment.

## 1 Introduction

Numerous models (e.g., cognitive mediational, athlete-coach relationship) have been developed to assess coaching and leadership effectiveness (1–4). While differences in these can be identified based on contextual factors and specific foci of each model, a general consensus is that successful outcomes for athletes and teams are influenced by the coaching style, athlete characteristics and the coach-athlete relationship (5). However, these assume time is available for the coach to build effective relations and to develop a long-term plan to help athletes grow and meet the desired goals. In tournament environments, where selected athletes are brought together to form a national team, this may not always be the case, making tournament coaching a unique environment with unique stressors (e.g., limited time, role clarity) for the coach and the team (6).

Salcinovic et al. (7), identified four key factors to successful team performance (1) leadership style—where better performance was found with open communication and transformative leadership, (2) performance feedback—where learning, reflection and positive reinforcement enhance performance, (3) supportive team behaviour—with clear team structure and strong group identity preferred, and (4) team orientation—with improved performance attributed to knowledge sharing, functional diversity and team cohesion. Underpinning all four factors is the need for effective communication.

Of the little research related to tournament coaching, Donoso-Morales, Bloom and Caron (8) found that planning and communicating expectations to be key coaching components necessary for success. At tournaments the time for preparation is significantly shorter than in regular season coaching, which increases the pressure on both coach and athlete (9). In many sports, particular futsal, tournaments are not common and major events only occur every two years. Therefore, there is a need to ensure that clear goals are created that allow each individual in the team to understand their role, focus on their objective and prioritise team development. Alongside planning, the coach needs strong communication skills, to ensure each athlete knows their role and the expectations of them (10). Within the time constraints that tournaments instill, the ability to build trust and team unity quickly is critical for success (6). It is also important for the coaching staff to manage their own emotions (i.e., using mindfulness) and keep their body language positive, as this will keep the athletes focused (8).

The limited research that has investigated women's sport teams has highlighted the need for empowerment coaching approaches to be crucial for successful performance (11). By allowing the team input into decisions and utilising a transformative leadership approach members become more motivated and can perform above expectation (7). Donoso-Morales et al. (8), noted that because the stakes are higher at a national championship more stress is perceived. For these coaches, implementing effective emotional management techniques was crucial for their team's success, with coaches specifically planning relaxation activities into the program (8). Management of emotions during the tournament was helped by setting performance goals that were within the athletes’ control.

Due to the minimal research available focusing on coaching at a tournament and coaching women at a sport tournament, the aim of this case study was to identify the actions and approaches a national women's team futsal coach employed during a week-long tournament. Of specific interest was how the coach managed the players, built team cohesion, and communicated with the playing group, and how these actions and approaches developed as the tournament progressed. Further, with the continual growth in women's futsal at international level, and the inaugural FIFA world cup in 2025, this knowledge can help other coach's and team to be better prepared for major events.

## 2 Methodology

### 2.1 Participant

The participant was appointed the head coach of a women's national futsal team a few months prior to taking the team to the Futsal Women's June Cup, a preparation tournament for the 2025 World Cup qualifying event to be held later in the year. She was a former professional player and member of the national team in both football and futsal. She had previous coaching experience in both women's and men's football and futsal but not at international tournaments, and as a physical education teacher. She was supported at the tournament by an assistant coach, physical conditioning coach, physiotherapist, and doctor (all women) and a goalkeeping coach (man). The tournament was played across a one-week period, with the team spending nine days living together in hotel accommodation. No further details are provided, to maintain some confidentiality for the participant and team.

### 2.2 Research design

An interpretive ethnographic case study design was utilized with the coach interview on separate occasions in the buildup to the tournament, during the tournament and after the tournament had finished. An interpretive paradigm aims to provide and understanding of the human experience (12), while ethnography allows for the phenomenon to be examine in its natural environment (13). The benefit of ethnography is allowing a holistic and immersive approach to research (14), and encourages continued engagement over time (15). The unique nature of tournament coaching makes this a valued methodological approach to gain the insights into processes and procedures that can fluctuate rapidly due to competition results and/or other coaching and non-coaching factors.

### 2.3 Research procedure

Semi-structured interviews were conducted by the first author with the coach on five separate occasions. An initial interview was completed before the tournament commenced, followed by three interviews during the course of the tournament, and finally a reflective interview was completed three days after the tournament finished. Guided by the contemporary literature, interview questions focused on the coach's approach to developing cohesion within the team, her approach to building relationships with each player, the process of working with other coaches and support staff, how coaching strategies were adapted between games, and reflections on challenges that occurred. Prior to the interviews an external subject specialist and research provided feedback on the interview guide to enhance quality. The aim of each interview was to capture how the coach worked with these athletes and managed the known time pressure, and how she adapted her coaching to meet the needs. Each interview was recorded and transcribed to produce 77 pages of single-line text that produced a detailed record of the lived experience.

### 2.4 Data analysis

Inductive, thematic coding, as suggested by Rivas (16), was employed by the first author on the interview transcripts, using Nvivo 13 software (Lumivero). Firstly, the transcripts were read and re-read to obtain familiarity with the content. During this process memos were created to identify information deemed important to the phenomenon being investigated. Next, a zigzagging strategy was implemented to identify initial codes, that guided subsequent data coding to refine the thematic codes. The transcripts and codes continued to be analysed until no new data emerged. *in vivo* codes, which keep the lived experience, using gerunds (17) were applied on a sentence-by-sentence basis, which helps to avoid misinterpretation. Next these codes were grouped into categories that were reviewed to form lower-order themes. These lower-themes were then grouped into distinct higher-order themes of the greatest generalisability. At each stage the other members of the research team reviewed and reflected on the emerging codes and themes.

## 3 Results

**Figure 1:**
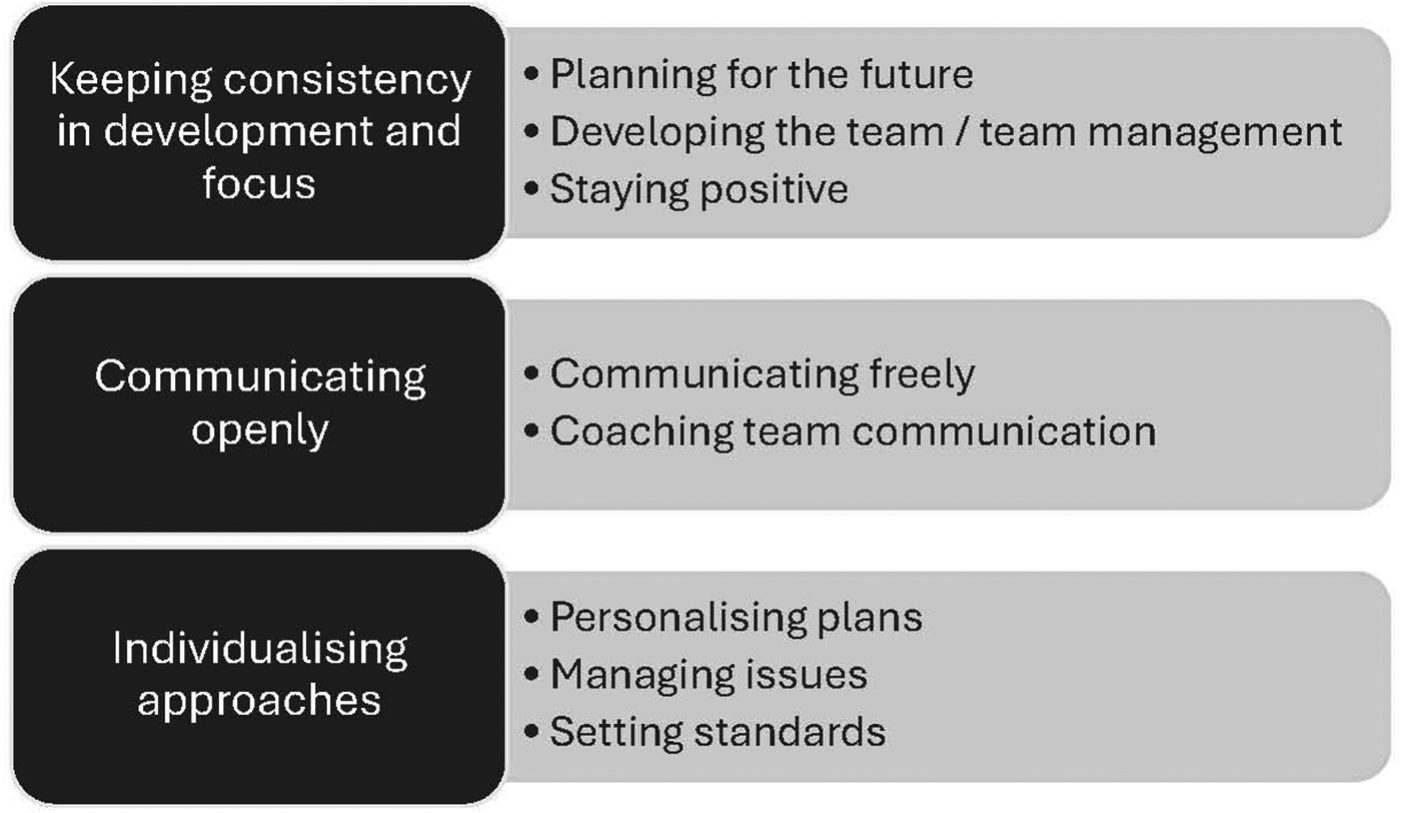
Higher-order and lower-order themes.

Three higher-order themes were identified during the thematic coding: (1) Keeping consistency in development and focus, (2) Communicating openly, and (3) Individualizing approaches. These were developed from eight lower-order themes that pertained directly to coaching this women's team. See Figure 1.

### 3.1 Keeping consistency in development and focus

A crucial component to the development of the team was to be consistent in what they were doing and focusing on. As a preparation event for the World Cup qualifiers later in the year, this tournament was focusing on the team's tactics and strategies, ability to work together, and how they could react to different game scenarios. The coach explained:

We are using this as an opportunity for all the players and we just we learn from the mistakes. I don't have problems with making mistakes. You need to continue trying what we are doing and telling you to do and that’s it.

This meant that the team tactics for each game stayed the same for each match, as the coach tried to implement her system to the playing group. As the tournament continued, the coach aimed to empower the senior players to provide input. “[older players, specifically player #1] is taking the responsibility. She is responding well. She is there for them. She's a good leader, along with maybe two or three other girls that are asking questions and are very intelligent in their communication”.

There were some specific strategies used to keep this consistency. Primarily a positive approach was taken in all areas, with the team focused on what had gone well and not dwelling on performance errors. This positivity allowed the players to concentrate on development and not worry about being called out for mistakes, as many of them had sometimes experienced in the past. The coach elaborated, “we are taking a much more focused, positive kind of thing and there doesn't need to be the more aggressive kind of style within what we're doing”. This encouraged the players to be more proactive and less anxious. As a result, the players appeared to become more empowered and would contribute further to the team development. It also allowed some of the younger players to become more integrated into the team, which was a crucial part of planning for the future. Similarly, the players were also more willing to accept decisions made by the coaching staff. For example, the decision to allocate players to rooms based on position was understood. The coach explained,

I was thinking a lot because I was doing the plan and the schedule for their rooms, together with the staff. And that’s something that we also did good because we changed a little bit. So, they can know each other and for example, the goalkeeper, who is a little bit older, she normally was in with her friend, but now we’ve put the goalkeepers together. The three goalkeepers together and on the first day it was “*Oh my God, I have to go with another goalkeeper*” … But yesterday at the individual meeting, she said that she is very positively surprised because they started to talk. They are building relationships.

Following the tournament, the coach reinforced this positive approach by “talking to the girls and I asking each of them to say what was the most valuable moment for each of them”. This reflection will form the base of the future team development activities.

### 3.2 Communicating openly

After playing under more autocratic male coaches in her playing career, the coach was aware that a more open and free communication style was required to engage the players and encourage them to play to their potential. She made time for players to speak in team meetings, which helped create a positive energy and calmness within the playing group, and increase cohesiveness. She explained, “their energy is very good. They are very calm. They feel like they can express themselves. They feel like they can ask question”. They continued to explain that it was important that, “I'm not here to forbid anything. I want them to choose what they want to do”. This open communication encouraged team development and autonomy. While it was difficult for the players who were not used to such an approach, to engage initially, they soon opened to the process and used this to develop the team culture and cohesiveness. This was enhanced after the tournament when the coach, “had a final meeting with them before leaving for home. You know before everybody goes on their way, to give them some goals for the future; What we expect from them or what is the idea of the national team”. This ensured that this tournament was not seen as just an individual occasion but rather part of a bigger picture and development towards the World Cup qualification.

The head coach was also responsible for the wider coaching and sport science team. Within this, she needed to ensure that these staff adopted her philosophical approach to the team. Key was “good communication” between them. She explained,

I would say the energy of my staff is very professional when we are working and very relaxed when we are off the pitch, and they are also in good communication with the girls. So, I think we are kind of transferring the good energy and the normal behavior towards them.

In particular she worked closely with her assistant coach and used this opportunity to help develop her [the assistant] coaching skills. “My assistant coach also is growing… She doesn't have a lot of coaching experience, so it is normal that sometimes she misses small things in communication and the coaching elements. But then when the timing is right, we talk to each other about what is important”. This was also a deliberate ploy to ensure the assistant's role at future tournaments could be enhanced.

### 3.3 Individualizing approaches

A deliberate effort was made by the coach to individualize plans for players. This included physical training, video feedback and personal planning sessions with each player. By doing so, the coach was able to further develop player autonomy and ownership and provide clear objectives for each player that was role specific. “I had individual meetings with them just to hear their feedback and how they feel”, the coach explained. She focused on this specifically because she thinks it was essential when working with women.

I think it is different in the way that when girls feel that they have support and they have trust in their coach, they give you more and they open even more, you know. Boys are like, they just want to play football. But girls need time to feel that they can express themselves and that’s what is happening when you are communicating fairly with them.

The positive results of this approach were easily observed by the coach, particularly in the younger players. She explained, “I had individual training sessions with six younger players. And when we isolated from the [older players], they were blossoming. Like they, they received the tactical information easily and they managed to do that in the game”.

The willingness of the coaching staff to be open to individual player needs, was helpful in building positive relations between the staff and players. The players were able to respect that the coaches understood their situation and that the coaching team was there to support the players. To develop the team culture further, the coaching team used the senior players to help set standards and behavioural norms.

## 4 Discussion

Within the refinements of a regular season, coaches have recognised the need to implement activities that are designed to better prepare their athletes for end of season championship tournaments (10). A similar approach was taken by the coach in the current study, as she aimed for consistency in development and focus with an eye on the more important World Cup qualifying tournament later in the year. While no research has assessed the effectiveness of this approach, anecdotal reports note that this is common for coaches in tournament settings. Keeping the focus on development and reducing the emphasis on the end result is highly valued by female athletes (18), and this helped the coach to develop a strong coach-athlete relationship. To achieve this the coach provided clear goals, expectations and behaviours for the players. Donoso-Morales et al. (10) have previously identified that this becomes even more critical in the tournament setting, especially as emotions can be heightened by the increases in stakes. During a tournament, supportive coaching behaviours can also enhance performance (19), with female athletes also valuing the development of transferrable skills and personal support (20).

For female athletes, coaches need to adapt their coaching to meet the athletes’ needs (21), with transformative coaching approaches appearing more beneficial in tournament settings (8). The coach in the current study made a conscious effort to do this by implementing an open approach to communicating with her players and other staff. Further research is required, but this could also be a method to ensure better athlete wellbeing (22) Stewart (18) noted that a positive coach-athlete relationship allows for more open communication, and despite the intensity of being a tournament the coach concentrated on positive outcomes and provided feedback that encouraged athlete autonomy. The open communication also ensured that each player and staff member understood their role and responsibility, which is essential to succeed in a tournament (6). The coach in the current study reflected on the importance of this approach to building cohesiveness within the staff.

An empowerment approach is beneficial for coaches to build team cohesion and encourage a quick acceptance of the team goals and culture (7). A key focus for the coach in the current study was to work with each player individually, building their capacity to perform at this level and create individualised development plans. Like Vallée and Bloom (11), this included both on and off court components. Individualising plans for female athletes is an important component of building coach-athlete relations (21). Within the tournament environment this is more challenging, as the head coach is also responsible for the planning and management of all activities at the tournament (6) and therefore, can be time challenged by other demands. However, coaches who are more structured and unwilling to adapt reduce female athletes’ willingness to participate (18). Similar ideas have been suggested for female athletes management of injury (23), and how to address gender environmental challenges faced by female athletes. As such, coaches need to be aware of the emotional climate of a tournament and adjust accordingly. One strategy employed by the coach in the current study was to use more experienced players to help reinforce standards and expected behaviours. While the effectiveness was not specifically measured, the coach recognised this to been well received by the players and important for development. Donoso-Morales et al. (8) noted that the use of other players to provide leadership is helpful in tournament environments. By doing so, the coach can create others to be role models for less experienced members of the team, which many female athletes desire (20).

## 5 Conclusion

There are significant challenges to coaching in a tournament that are not present in general coaching settings. The time constraints require coaches to plan in detail and clearly communicate with athletes and support staff. The current study adds to the developing knowledge of how coaches manage this, identifying three key strategies: keeping consistency in development and focus; communicating openly; and individualising approaches. The development of positive coach-athlete relations, where the dyad has mutual trust, aids the process. This appears especially true for female athletes. The coach needs to adapt to what is happening during the tournament and understand how this may influence the emotional climate for the athletes. For the coach in the current study, staying positive and empowering her players to develop autonomy allowed for success. While this can be more difficult at a tournament, it is essential that the coach understands this and adapt their behaviour to meet the athlete needs.

## Data Availability

The datasets presented in this article are not readily available because there is a limited population that the data could come from and in order to keep some confidentiality for the participants the data is restricted, but an edited version can be requested. Requests to access the datasets should be directed to fcarson@lunex.lu.
